# Hormone biosynthesis and metabolism members of 2OGD superfamily are involved in berry development and respond to MeJA and ABA treatment of *Vitis vinifera* L

**DOI:** 10.1186/s12870-022-03810-7

**Published:** 2022-09-06

**Authors:** Yingying Gao, Xiaochen Wang, Xianju Liu, Zhenchang Liang

**Affiliations:** 1 Beijing Key Laboratory of Grape Science and Enology, CAS Key Laboratory of Plant Resources, Institute of Botany, The Chinese Academy of Sciences, Beijing, 100093 China; 2 University of Chinese Academy of Sciences, Beijing, 100049 China; 3 Key Laboratory of Beijing for Identification and Safety Evaluation of Chinese Medicine, Institute of Chinese Materia Medica, China Academy of Chinese Medical Sciences, Beijing, 100700 China

**Keywords:** 2OGD superfamily; LBO; hormone; ripe; grape

## Abstract

**Background:**

Hormones play an indispensable role during fruit ripening, nine clades in 2-oxoglutarate-dependent dioxygenase (2OGD) superfamily are responsible for the hormone biosynthesis and metabolism, but less information is known about them.

**Results:**

A total of 163 *Vv2OGD* superfamily members were identified from grape genome, which were mainly expanded by local (tandem and proximal) duplication. Phylogenetic analysis of 2OGD members in grape and *Arabidopsis* indicates 37 members in Vv2OGD superfamily are related to hormone biosynthesis and metabolism process (*Vv2OGD-H*), which could be divided into 9 clades, gibberellin (GA) 3-oxidase (GA3ox), GA 20-oxidase (GA20ox), carbon-19 GA 2-oxidase (C_19_-GA2ox), carbon-20 GA 2-oxidase (C_20_-GA2ox), 1-aminocyclopropane-1-carboxylic acid oxidase (ACO), dioxygenase for auxin oxidation (DAO), lateral branching oxidoreductas (LBO), downy mildew resistant 6 and DMR6-like oxygenase (DMR6/DLO) and jasmonate-induced oxygenase (JOX). Sixteen of these 37 *Vv2OGD-Hs* are expressed in grape berry, in which the expression patterns of *VvGA2oxs*, *VvDAOs* and *VvJOXs* shows a correlation with the change patterns of GAs, indole-3-acetic acid (IAA) and jasmonates (JAs), indicating the involvement of these genes in grape berry development by regulating corresponding hormones. Twelve *Vv2OGD-Hs* respond to methyl JA (MeJA) treatment, of which eight may lead to the inhibition of the ripening process by the crosstalk of JAs-salicylic acids (SAs), JAs-GAs and JAs-JAs, while seven *Vv2OGD-Hs* respond to ABA treatment may be responsible for the promotion of ripening process by the interplay of abscisic acid (ABA)-strigolactones (SLs), ABA-SAs, ABA-GAs, ABA-JAs. Especially, *VvLBO1* reach an expression peak near véraison and up-regulate about four times after ABA treatment, which implies SLs and ABA-SLs crosstalk may be related to the onset of berry ripening in grape.

**Conclusions:**

This study provides valuable clues and new insights for the mechanism research of *Vv2OGD-Hs* in hormones regulation during the grape berry development.

**Supplementary Information:**

The online version contains supplementary material available at 10.1186/s12870-022-03810-7.

## Background

The 2-oxoglutarate-dependent dioxygenase (2OGD) superfamily widely exists in eukaryotes and bacteria, it catalyzes the oxidation reaction during the formation of many metabolites in organisms [1]. Most of the 2OGD enzymes work as follows: the substrate is oxidized under the existence of 2-oxoglutarate (2OG) and molecular oxygen, this process is catalyzed by Fe (II) cofactor and 2OGD, then 2OG decarboxylation and forms succinic acid and carbon dioxide after the reaction (2OG + O2 + S → succinate + CO2 + SO, S represents the substrate). Structural analysis shows all reported 2OGD enzymes contain a double-stranded β- helix (DSBH) folding, which provides a scaffold for Fe^2+^ binding. At one end of the DSBH, it has a conservative site [His-Xaa-Asp/Glu-(Xaa)n-His] for Fe^2+^ binding among 2OGD members. Except that, there is a less conservative [Arg-Xaa-Ser/Thr] motif whose function is still not clear [2]. 2OGD superfamily could be divided into three classes based on the evolutionary tree: DOXA, DOXB and DOXC [3]. DOXA class is composed of AlkB homologs (ALKBH), ALKBH in plants originates from the ALkB in prokaryotes which plays an important role in DNA repair [4, 5]. The members in DOXB are P4Hs, they participate in the post-translational modification of protein and hydroxylate proline residues by forming 4-hydroxyproline [3]. DOXC is the largest branch of 2OGD, with 99 and 159 members in *Arabidopsis* and tomato respectively [6]. Until now, DOXC members have been found to be involved in hormone biosynthesis or metabolism, and catalyze the synthesis of some secondary metabolites, such as flavonoids, benzylisoquinoline alkaloids, glucosinolates and tropane alkaloids, etc. [1, 2].

Hormones play indispensable roles during plant growth and development. According to the evolutionary tree and gene function, the hormone biosynthesis and metabolism related members in DOXC class (abbreviated as 2OGD-H below) could be divided into 9 clades [6]. **1) Gibberellin (GA) 20-oxidase (GA20ox)** catalyzes two steps in two GA biosynthesis pathways respectively, GA12 → GA24 → GA9 and GA53 → GA19 → GA20. The products GA9 and GA20 are precursors of active GAs. In rice and *Arabidopsis*, knock-out of *GA20ox* lead to dwarfed statures, on the contrary, its overexpression leads to elonged phenotype [7, 8]. Subsequently, **2) GA 3-oxidase (GA3ox)** catalyzes the precursors GA9 and GA20 to finally form active forms GA4 and GA7, GA1 and GA3, respectively. The dwarfed phenotype of *GA3ox* deletion in *Arabidopsis* is similar to that of *GA20ox* [9]*.* However, GA 2-oxidase (GA2ox) oxidizes active GAs or their precursors to inactive forms. GA2ox contains two clades **3)** Carbon-19 GA 2-oxidase **(C**_**19**_**-GA2ox)** and **4)** carbon-20 GA 2-oxidase **(C**_**20**_**-GA2ox)** regarded as their different substrates carbon-19 (C_19_)-GA and C_20_-GA [3]. Correspondingly, overexpression of *GA2ox* in rice leads to dominant dwarf and GA deficient phenotypes, which are opposite to *GA20ox* and *GA3ox* [10]. **5) Dioxygenase for auxin oxidation (DAO)** causes the oxidative inactivation of indole-3-acetic acid (IAA), rice mutants *dao* showed an increased level of free IAA in anthers and ovaries [11]. **6) 1-Aminocyclopropane-1-carboxylic acid (ACC) oxidase (ACO)** catalyzes the oxidation of ACC to ethylene (ET), which is functional in many aspects of plant growth by limiting the rate of ET release, such as stress resistance, maturity, flower development and gender determination, etc. [12] **7) Jasmonate (JA)-induced oxygenase (JOX)** hydroxylated JA to an inactive form, the deletion of four *JOXs* in *Arabidopsis* could up-regulate the defensive genes expression and enhance the resistance to necrotrophic fungus *Botrytis cinerea* and caterpillar *Mamestra brassicae* [13]. **8) Downy Mildew Resistant6 and DMR6-like oxygenase (DMR6/DLO)** catalyzes the inactivation of salicylic acid (SA) by hydroxylating it. DMR6 acts as an SA-5-hydroxylase (S5H) enzyme while DLO acts as an S3H enzyme, they transform the active SA into 2,5-dihydrobenzoic acid (DHBA) and 2,3-DHBA respectively. Studies on *Arabidopsis* overexpression lines and mutants suggest *DMR6/DLO* genes could reduce stress resistance and immunity of plants [14, 15]. **9) Lateral branching oxidoreductase (LBO)** catalyzes MeCLA (one of the active form of SLs) to [MeCLA + 16 Da], a more active strigolactone (SL) form [16]. Compared with wild type, branches number of *Arabidopsis lbo* mutant is increased [17].

Grapevine (*Vitis vinifera* L.) is one of the most popular fruit trees in the world, it can be used as fresh food, or as materials for wine, juice and raisin, which has huge economic value. It is well known that phytohormones are important regulators for berry growth and the development of grapes. IAA, JAs, cytokinins (CKs), SAs and GAs have high levels in the early stage of grape berry development, then decrease gradually with the fruit ripening, these hormones were defined as putative ripening inhibitors in some studies [18–20]. The peaks of abscisic acid (ABA) and ET appear around the véraison stage, these two hormones usually are considered to be related to the onset of berry ripening [19, 20]. In agricultural production, it is one of the most economical and convenient methods to regulate fruit ripening and quality with exogenous hormones application. For example, ABA is the most commonly used hormone to promote ripening in grapes, the processes of berry softening, anthocyanin, and sugar accumulation were accelerated after exogenous ABA treatment [19, 21, 22]. Auxin was considered as the most frequently used and effective ripening inhibitor in grape production [18], treating the grape bunches with 1-naphthaleneacetic acid (NAA) at pre-véraison stage delayed technological maturity by ~ 30 days, while the quality of ripening fruit was not affected [23]. But interestingly, it was recently reported spaying MeJA to leaves and fruit clusters could also lead to a delay in grape ripening, meanwhile, the concentration of monoterpenes increased significantly [24] — which confer typical floral notes to *Muscat* cultivars. Thus, MeJA may have a more promising prospect as a ripening inhibitor in future. There is complex crosstalk of ABA or JAs with other phytohormones in *Arabidopsis* and rice [25–28], since that, is the crosstalk also exists in the process of ABA or MeJA promoting or inhibiting grape ripening? Whether the hormones crosstalk is conservative or differential in grape berry compared with *Arabidopsis* and rice? The expression changes of *Vv2OGD-Hs* may provide some useful information.

Some of the 2OGD-H members have been reported to be involved in the ripening process, such as GA2ox, ACO, and DAO [6, 12, 29]. However, most of the members whether functional in ripening have not been studied, such as JOX, DMR6 and LBO, meanwhile, the related hormones JAs, SAs and SLs have already been researched and show significant involvement in berry development or quality formation [18–20, 29, 30]. In grapes, 2OGD superfamily members are still not identified from a genome-wide level except GA oxidase enzymes [29, 31]. In this study, the grape 2OGD superfamily was identified using the published genome [32]. Then the members related to hormone biosynthesis and metabolism (2OGD-H) were picked out based on the evolutionary tree, and their functions were predicted through amino acid sequence alignment, motif analysis and duplication analysis. Besides, the expression patterns during berry development were studied using published transcriptome data [33], and the ripening inhibitor — MeJA, and promoter — ABA have been applied to *Vitis vinifera cv.* ‘Jingxiangyu’ at the pre-veraison stage, aiming to investigate *Vv2ODG-H* genes’ response to ABA and MeJA. Finally, several Vv2OGD-H members were obtained, which may play significant roles in the berry development by regulating corresponding hormones or hormone crosstalk. This study will provide valuable clues and new insight for the investigation of hormone regulation mechanisms during grape berry development.

## Results

### Identification of 2OGD superfamily members and screening of 2OGD-H enzymes in grape

**Fig. 1:**
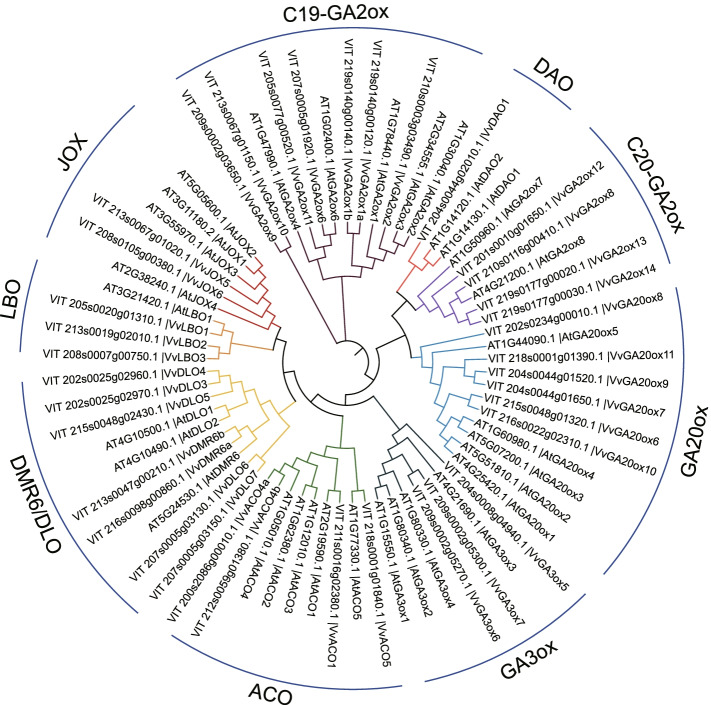
Evolutionary tree of hormone biosynthesis and metabolism members of 2OGD superfamily in *Arabidopsis* (31) [3] and grape (37). The branches in different clades are distinguished in different colors

A total of 163 putative 2OGD superfamily members were identified from the grape genome, the detailed information is summarized in Suppl. Table S1. The grape 2OGDs identified above were used to construct the evolutionary tree with *Arabidopsis* 2OGDs [3] (Suppl. Fig. S1). According to the tree, Vv2OGDs can be divided into three classes: DOXA, DOXB and DOXC, which contain 7, 9, and 146 members, respectively. Except that, there is one member *VIT_201s0011g06280.1* could not be clustered with any class. Further, DOXA, DOXB and DOXC classes were divided into 6, 5 and 25 clades based on the previous study [3]. Among them, DOXC-31 clade and DOXC-52 clade have the most members, 25 and 26 respectively, while some clades contain only one member, such as DOXA-3, DOXB-5, DOXC-14, etc. (Suppl. Fig. S1, Suppl. Table S1). Partial Vv2OGDs were named based on functional At2OGDs names and the published gene nomenclature system in grape [34]. Nine clades in DOXC class are related to hormone biosynthesis and metabolism with 37 members in total, which are 6 members in GA20ox, 3 in GA3ox, 7 in C_19_-GA2ox, 4 in C_20_-GA2ox, 1 in DAO, 4 in ACO, 2 in JOX, 7 in DMR6/DLO and 3 in LBO (Fig. 1, Suppl. Table S1).

### Gene duplication, chromosome distribution and collinearity analysis of Vv2OGDs

**Fig. 2:**
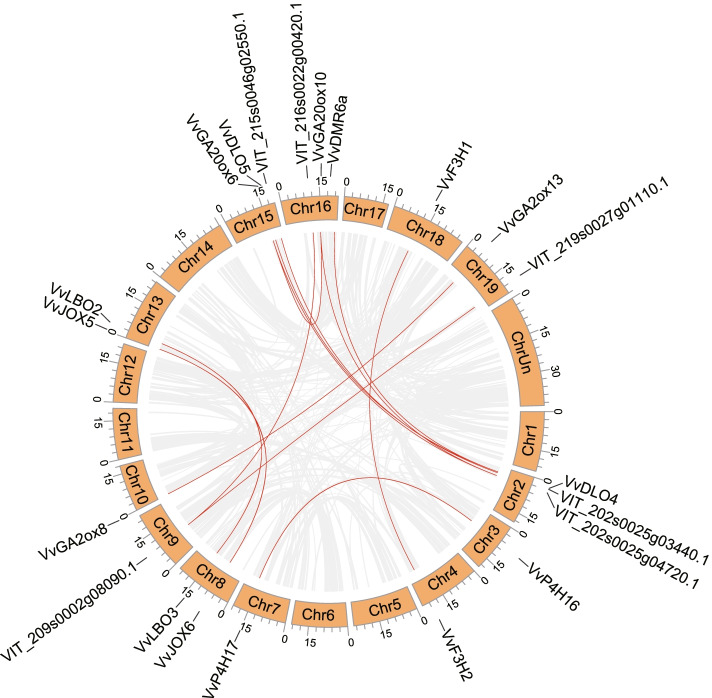
WGD/Segmental duplication analysis of *Vv2OGD* superfamily. Only *Vv2OGDs* involved in the WGD event and their distributed chromosome are shown. Chromosomes of grape are shown in one orange circle with indicated numbers in it. The location of *Vv2OGDs* is shown by short black lines beside the circle. Gray links in the circle represent all WGD duplication that occurred in grape genome, while the red represents WGD involved in *Vv2OGD* superfamily

**Fig. 3:**
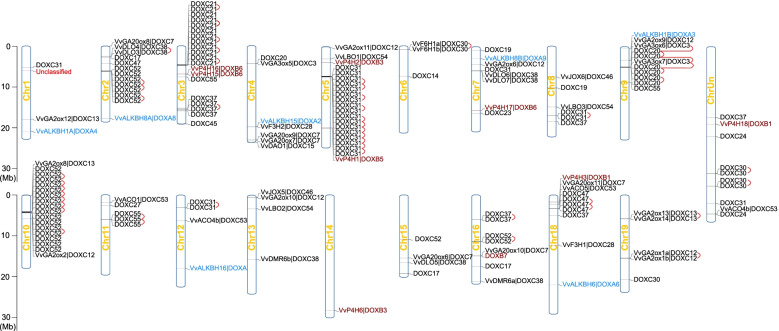
Chromosome distribution and tandem duplication analysis of *Vv2OGD* superfamily. The bars with blue lines represent chromosomes, each *Vv2OGDs* are marked on it with gene name and belonging clade (the genes with unidentified functions and names only indicates the belonging clade). *Vv2OGDs* in DOXA, DOXB and DOXC classes are distinguished by blue, red and black font, respectively. Tandem duplications in *Vv2OGDs* are indicated by red curves

There are four gene duplication ways in angiosperms: whole-genome duplication (WGD, also called segmental duplication), tandem duplication, proximal duplication and dispersed duplication [35]. 12.9% of *Vv2OGDs* are replicated by WGD, which is 21 *Vv2OGDs* involved in 13 WGD events (Fig. 2), most of them produce the same clade genes after WGD, such as *VvLBO2*-*VvLBO3*, *VvGA2ox8*-*VvGA2ox13*, *VvJOX5*-*VvJOX6*, *VvGA20ox6*-*VvGA20ox10*, *VvDLO4*-*VvDLO5*, *VvDMR6a*-*VvDLO4*, *VvF3H1*-*VvF3H2* and *VvP4H16*-*VvP4H17*. As shown in Fig. 3, 73 *Vv2OGDs* are involved in tandem duplication events, accounting for 44.8%, and all of them form the same clades genes after the duplication. Except that, there are 26 genes duplicated by the proximal way, accounting for 16.0% (Suppl. Table S1). It can be seen from the above that local duplication (tandem duplication and proximal duplication) is the major way for *Vv2OGDs* superfamily expansion. Especially, all members of DOXC-21 on chromosome 3, DOXC-52 members on chromosome 2 and chromosome 10 are formed by local duplication. Figure 3 also shows the distribution of the *Vv2OGD* family on chromosomes. *Vv2OGDs* distribute unevenly on all chromosomes except for chromosome 17. There are more *Vv2OGD* genes on chromosomes 2, 3, 5, 9 and 10 and they form gene clusters, while some chromosomes contain fewer *Vv2OGDs*, such as chromosome 14 only owning one *Vv2OGD*.

**Fig. 4:**
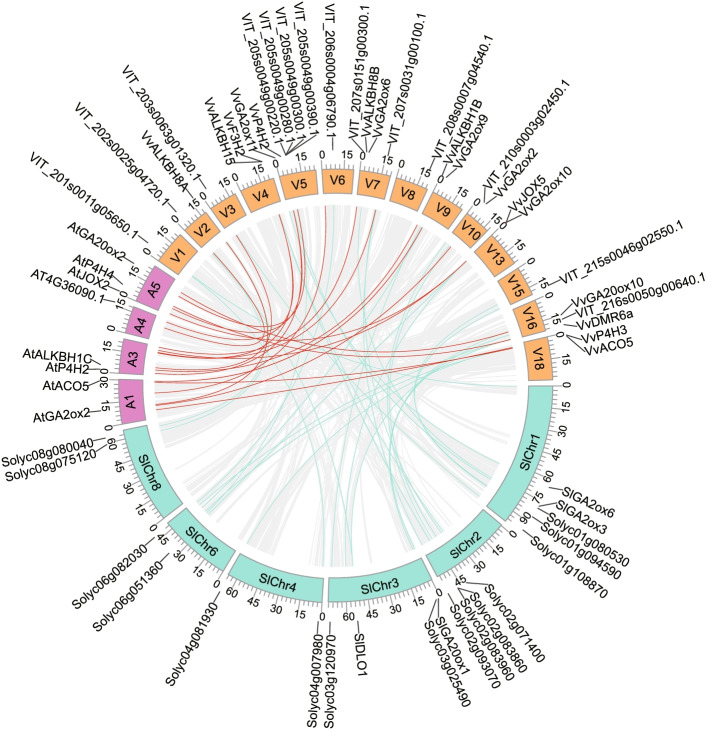
Collinearity analysis of *2OGD* superfamily members in grape, *Arabidopsis* and tomato. Only *2OGDs* involved in the collinearity region and their distributed chromosome are shown. Chromosomes of grape, *Arabidopsis* and tomato are drawn in one circle with indicated numbers in it and distinguished by orange, purple and green, respectively. The location of *2OGD* genes is shown by short black lines beside the circle. Gray links in the circle represent all collinearity regions in grape-*Arabidopsis* and grape-tomato. Red links represent collinearity *2OGD* gene pairs between grape and *Arabidopsis*, while green represents that between grape and tomato

Figure 4 shows the collinearity analysis results of *2OGD* members among grape, *Arabidopsis* and tomato, the details are listed in Suppl. Table S1. There are 26 collinearity pairs of 2OG members between grape and tomato, but only 8 between grape and *Arabidopsis*, which may be due to the closer phylogenetic relationship between tomato and grape [36]. Some collinear gene pairs belong to the same clade, such as *VvACO5*-*AtACO5*, *VvALKBH1B*-*AtALKBH1C*, *VvJOX5*-*AtJOX2*, *VvGA20ox10*-*AtGA20ox2*-*SlGA20ox1*, *VvGA2ox2*-*AtGA2ox2*, *VvGA2ox6*-*SlGA2ox3*, *VvGA2ox11*-*SlGA2ox6*, *VvP4H2*-*AtP4H2*-*AtP4H4*-*SlP4H*, *VvP4H3*-*SlP4H*, *VvF3H2*-*SlF3H*, *VvDMR6a*-*SlDLO1*, these genes may have conservative function among grape, tomato and *Arabidopsis*. Several gene pairs from different clades may come into different functions after the species' evolution.

### Sequence alignment and motif analysis of Vv2OGD-Hs

To explore whether the 37 Vv2OGD-H members are functional, sequence alignment was performed using the Clustal method to investigate the 2OGD family conserved sites of these 37 Vv2OGD-Hs. As shown in Suppl. Fig. S2, except VvGA2ox9, VvGA2ox10, VvGA2ox13 and VvDLO6, 33 members contain Fe (II) binding sites [His-Xaa-Asp/Glu-(Xaa)n-His] [3]. In addition, VvGA2ox9, VvGA2ox10 and VvGA2ox13 lack the motif of Arg-Xaa-Ser/Thr [2].

**Fig. 5:**
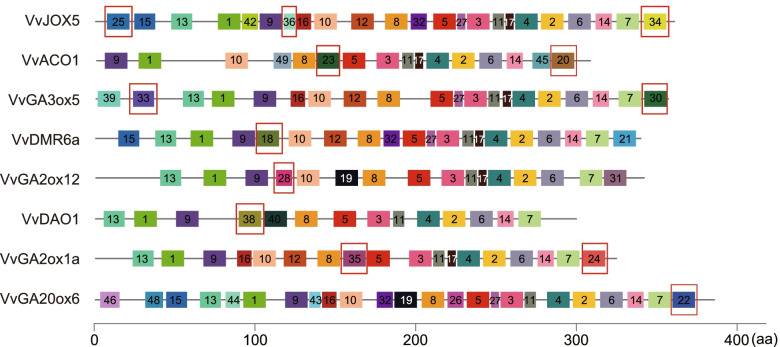
Represent motif analysis result of each clade of hormone biosynthetic and metabolism Vv2OGD proteins. The motifs specific to each clade are emphasized by red boxes. The sequence of each motif is listed in Suppl. Table S3

To further analyze the function conservation and differentiation, motif analysis was carried out on 31 At2OGD-Hs [6] and 37 Vv2OGD-Hs (Suppl. Fig. S3). It was found that most clades have clade-unique motifs (Fig. 5). For example, motif 25, 34 and 36 only exist in JOX clade, motif 20 and 23 only appear in ACO clade, GA3ox clade contains motif 30 and 33 uniquely, while motif 18, 28 and 38 specifically exist in DMR6, C_20_-GA2ox and DAO clade, respectively. All members in C_19_-GA2ox clade include motif 24 and motif 35 except VvGA2ox9 and VvGA2ox10. In GA20ox clade, except VvGA20ox8, other members all contain motif 22. But no motif specific to LBO clade was found.

### Expression patterns of *Vv2OGD-Hs* during grape berry development

As is described in the background section above, the 37 *Vv2OGD-Hs* are functional in six hormones biosynthesis and metabolism, GAs, IAA, ET, JAs, SAs and SLs [6]. Previous studies have proved that these six hormones play irreplaceable roles in fruit ripening or fruit quality formation process [18–20, 29, 30]. To understand whether the 37 *Vv2OGD-H* members were directly involved in berry development, their expression patterns were investigated by using the published transcriptome data (GSE98923) [33].

**Fig. 6:**
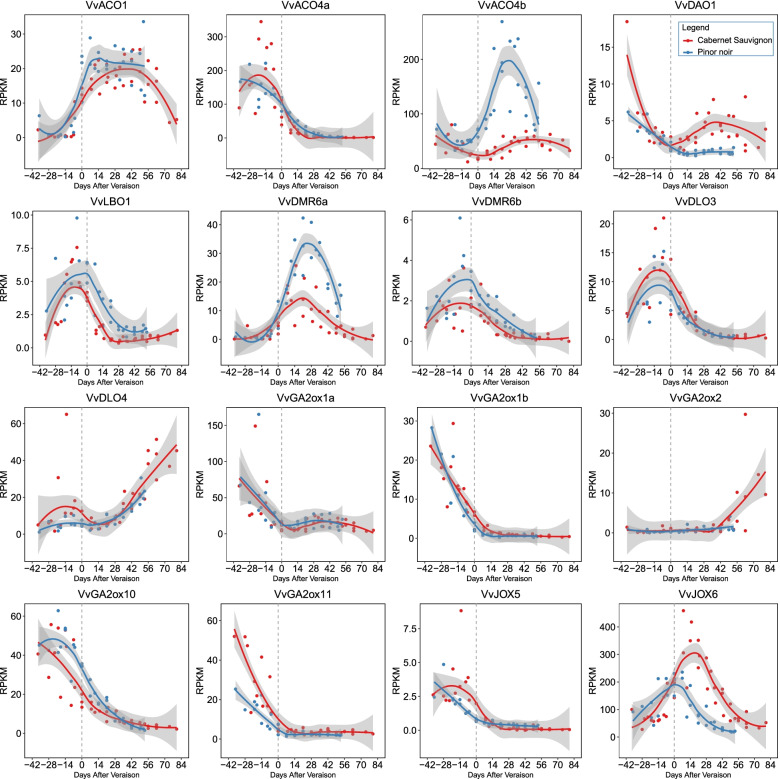
Expression patterns of *Vv2OGD-Hs* during berry development in ‘Cabernet Sauvignon’ and ‘Pinot Noir’. The plot graphs are drawn using data from three vintages (GSE98923) [33], the horizontal ordinate means days after véraison. Line graphs are the trendline of the plot and created by R, gray shading indicates 0.95 confidence levels relative to the smoothed conditional means plotting method

There are great differences among the expression patterns of different clades *Vv2OGD-Hs* in *Vitis vinifera* cv. ‘Cabernet Sauvignon’ and ‘Pinot Noir’. All members in GA20ox, C_20_-GA2ox and GA3ox clades almost have no expression in grape berry. Except that, 16 of the 37 *Vv2OGD-H* genes express during berry development (Fig. 6). Three *VvACOs* expressed in grape berry, *VvACO1* and *VvACO4b* have a low expression at fruit set stage then increase with berry development and reached highest after véraison, interestingly *VvACO4b* has a much higher RPKM value than *VvACO1*. On the contrary, *VvACO4a* is mainly expressed before véraison and gradually decreases with the ripening process. *VvDAO1* is highly expressed at fruit set stage, then decreases gradually and maintains a low expression level after the véraison stage. In LBO clade, only *VvLBO1* is expressed in berry, with an expression peak before véraison and then gradually down-regulated. Four members in DMR6/DLO clade express that their change patterns are different. *VvDMR6b* and *VvDLO3* have an expression peak before the véraison stage and then gradually down-regulated to an undetectable level before ripening, while *VvDMR6a* had a peak after the véraison stage, and *VvDLO4* was continuously up-regulated after véraison then reached the highest at maturity stage. Five members from C_19_-GA2ox clade, *VvGA2ox1a*, *VvGA2ox1b*, *VvGA2ox2*, *VvGA2ox10* and *VvGA2ox11* express during the fruit development, four of them (except *VvGA2ox2*) have highly accordant expression patterns, which is highly expressed at the beginning of fruit set, then significantly down-regulated with the berry growth to almost no expression after véraison stage. *VvJOX5* has an expression peak at the early stage of berry development then decreases to an undetectable level gradually, by contract, *VvJOX6* expresses much stronger and reaches the peak around the véraison stage.

### Analysis of *Vv2OGD-Hs* response to MeJA and ABA treatment

**Fig. 7:**
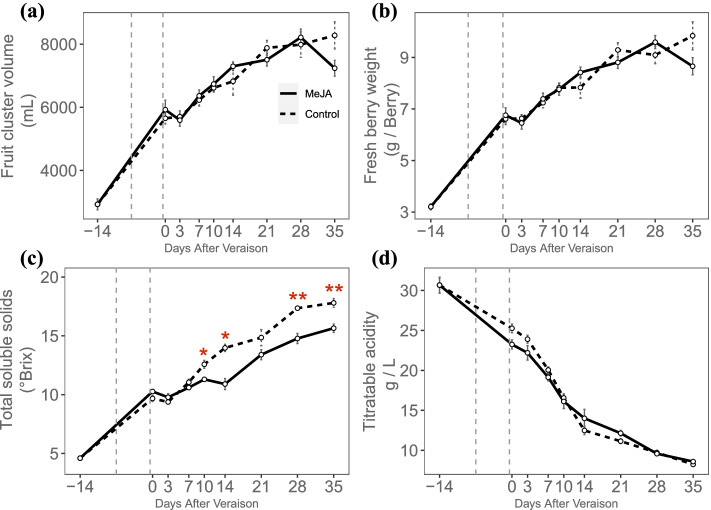
Physiological phenotype of ‘Jingxiangyu’ fruit after MeJA treatment. **a** The volume of fruit cluster. **b** Fresh berry weight. **c** The total soluble solids. **d** The titratable acid. The significant differences between control and MeJA treatment were analyzed by T-test using SPSS 20, and marked by “*” (*P* < 0.05) or “**” (*P* < 0.01)

**Fig. 8:**
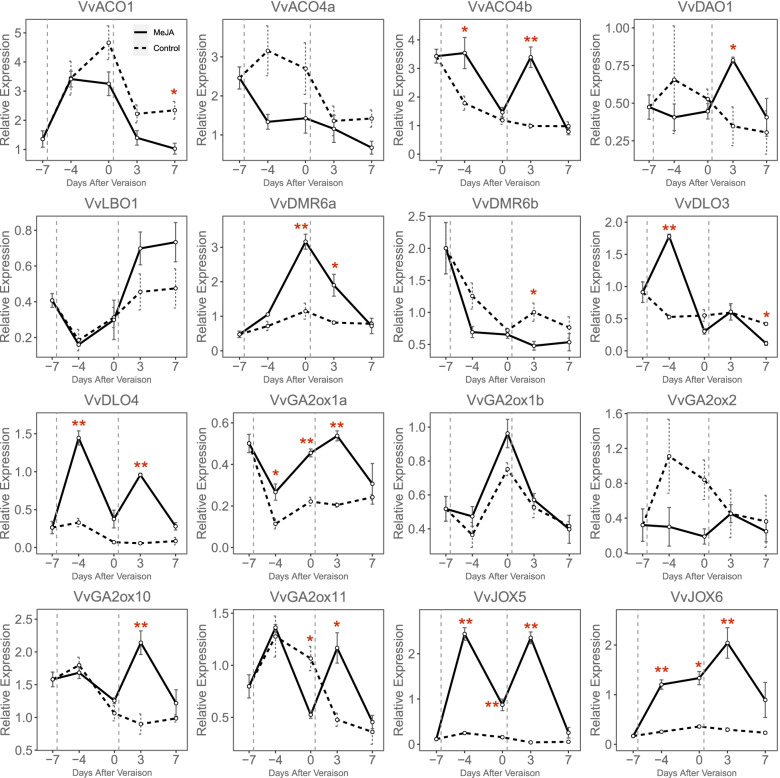
qRT-PCR results of *Vv2OGD-Hs* after MeJA treatment of ‘Jingxiangyu’ fruit. The horizontal ordinate means days after véraison. Vertical gray dotted lines represent the time points of the two treatments. The significant differences between control and MeJA treatment were analyzed by T-test using SPSS 20, and marked by “*” (*P* < 0.05) or “**” (*P* < 0.01)

The crosstalk of different hormones generally exists in plants, especially in the fruit ripening process [37]. To investigate the response to MeJA and ABA treatment of *Vv2OGD-Hs*, fruit clusters of *Vitis vinifera* cv. ‘Jingxiangyu’ were treated with MeJA or ABA two times, the first treatment one week before véraison, 7 days later fruits entered véraison stage and the second treatment was performed. As shown in Fig. 7, total soluble solid accumulate slower after MeJA treatment, while the berry cluster volume, single berry weight and titratable acid have no significant differences compared to control. Sixteen of the 37 *Vv2OGD-Hs* express in berry as described above (Fig. 6), further qRT-PCR result shows 12 of the 16 *Vv2OGD-Hs* significantly respond to MeJA treatment (Fig. 8). *VvJOX5* and *VvJOX6* have the strongest response. The expression of *VvJOX5* up-regulated about 88 times 3 days after the second treatment, meanwhile *VvJOX6* up-regulated about 7 times. Except that, the expression level of *VvACO4b*, *VvDMR6a*, *VvDLO3*, *VvDLO4*, *VvGA2ox1a*, *VvGA2ox10* and *VvGA2ox11* increase significantly after the first or second MeJA treatment, while *VvACO1*, *VvACO4a*, and *VvDMR6b* are down-regulated after two times MeJA treatments.

**Fig. 9:**
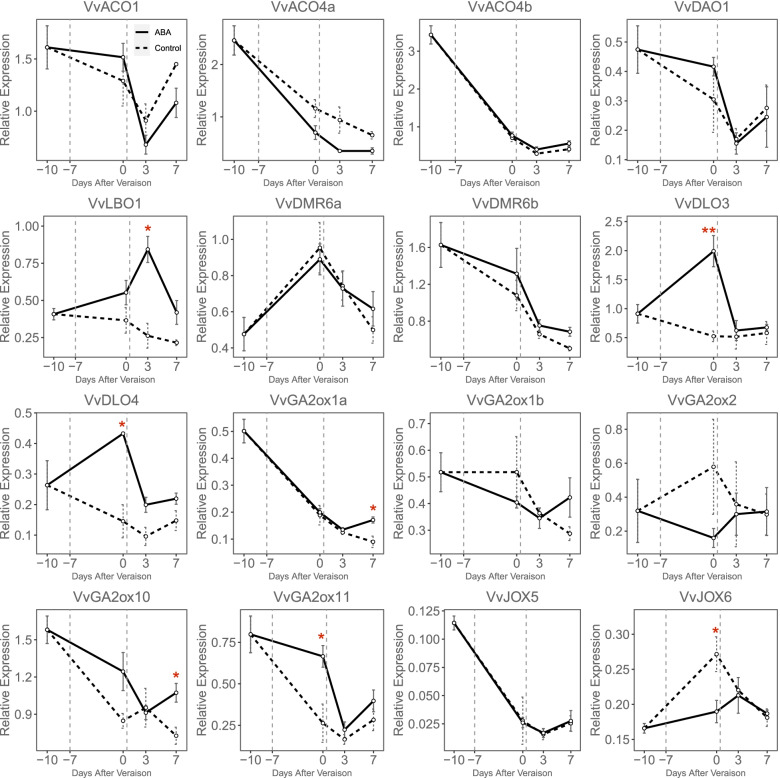
qRT-PCR results of *Vv2OGD-Hs* after ABA treatment of ‘Jingxiangyu’ fruit. The horizontal ordinate means days after véraison. Vertical gray dotted lines represent the time points of the two treatments. The significant differences between control and ABA treatment were analyzed by T-test using SPSS 20, and marked by “*” (*P* < 0.05) or “**” (*P* < 0.01)

The response to ABA treatment of *Vv2OGD-Hs* was investigated using ‘Jingxiangyu’ berry, the degradation rate of titratable acid is improved after ABA treatment (Wang et al., unpublished data of our lab). As shown in Fig. 9, the expression of seven *Vv2OGD-H* members is significantly changed after ABA treatment. *VvLBO1*, *VvDLO3*, *VvDLO4*, *VvGA2ox1a*, *VvGA2ox10* and *VvGA2ox11* are up-regulated after ABA treatment, while *VvJOX6* are down-regulated. Based on the above results, 16 *Vv2OGD-Hs* respond to MeJA treatment more dramatically than ABA, and it should be noted that the expression of *VvDLO3* and *VvDLO4* are both increased after the treatment of MeJA and ABA.

## Discussion

### Identification of Vv2OGD superfamily members

Kawai et al. first identified the 2OGD superfamily in *Arabidopsis*, rice, *Picea abies*, *Selaginella moellendorffi*, *Physcomitrella patens* and *Chlamydomonas reinhardtii*, which own 130, 114, 142, 74, 66 and 41 members respectively [3]. Followed by the 2OGDs identification in corn [38], tomato [6], *Salvia miltiorrhiza* [39], *Brassica rapa*, *Brassica oleracea* and *Brassica napus* [40], which have 103, 159, 132, 160, 179 and 337 members respectively. In this study, 163 *Vv2OGD* members were identified in grape, which is the third-biggest 2OGD family in identified species, only after *Brassica oleracea* and *Brassica napus*. According to the evolutionary tree and previous studies [3], the Vv2OGD members could be divided into three classes: DOXA, DOXB and DOXC, with 7, 9 and 146 members, and further are divided into 6, 5 and 25 clades, respectively (Suppl. Fig. S1, Table S1). Gene duplication analysis shows that local duplication contributes most to the expansion of *Vv2OGD* family (Fig. 2, Suppl. Table S1), but in maize, WGD/segment duplication is the predominant way for the 2OGD family’s expansion [38], which may result from the difference between monocotyledons and dicotyledons. Then, partial Vv2OGDs were named based on the evolutionary relationship with identified functional At2OGDs in the phylogeny tree, 37 Vv2OGD members in total are putatively related to hormone biosynthesis and metabolism (Suppl. Table S1, Fig. 1).

The characteristic conserved sequences of reported 2OGDs include a Fe (II) binding site [His-Xaa-Asp/Glu-(Xaa)n-His] located in the DSBH folding region and a [Arg-Xaa-Ser/Thr] motif whose function is unknown yet [2, 3]. Among 37 Vv2OGD-H enzymes, VvGA2ox9, VvGA2ox10, VvGA2ox13 and VvDLO6 lack the [His-Xaa-Asp/Glu-(Xaa)n-His] or Arg-Xaa-Ser/Thr motif (Suppl. Fig. S2), in which the expression of *VvGA2ox9*, *VvGA2ox13* and *VvDLO6* could not be detected in grape fruit (Fig. 6), thus these members may lose their function after family expansion. Further, the clade-unique motifs were obtained by motif analysis using MEME [41] (Fig. 5), which may result in the different functions of 2OGD-H enzymes in different clades, but this conjecture may need to be verified by more evidence.

### Vv2OGD-H genes may be involved in grape berry development by regulating endogenous hormones

**Fig. 10:**
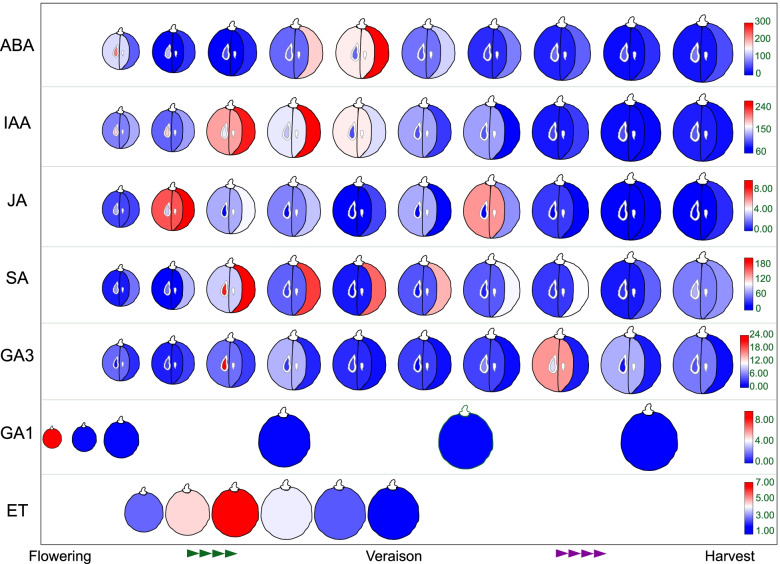
Diagram of the changes in six endogenous hormones with the berry development of Cabernet Sauvignon. The content data of ABA, IAA, JA, SA and GA3 in pericarp, flesh and seed were obtained from the previous research of Gao et al. [20], GA1 in deseed berry was from Symons et al. [43], while the changes data of internal ET was from Chervin et al. [42] Different colors were used to represent different content level as the legend indicates, the content unites of ABA, IAA, JA, SA, GA3 and GA1 are ng/g FW (fresh weight), while the ET content unite is pmol/min/g FW

Among the 37 *Vv2OGD-Hs*, 16 are expressed in grape berry (Fig. 6), and the published hormone changes data (Fig. 10) [20, 42, 43] are used to conjecture if they are involved in the berry development process by regulating corresponding hormones. ACOs, GA oxidases and DAO have already been reported functional during the berry development [6, 12, 29]. The previous research confirmed there is a small but clear ethylene peak around the véraison stage in three grape cultivars, and the expression peak of *VvACO4a* (called *VvACO1* in reference) coincided with the ethylene peak (Fig. 10) [42, 44, 45]. In this study, *VvACO4a’s* expression peak appeared about 2 weeks before véraison (Fig. 6), which is similar to the previous study, this result further proved the possible leading role of *VvACO4a* in ACO clade in ethylene biosynthesis of grape berry. IAA has the highest level in flowers and young fruits of grapes, then gradually decreases to a lower level with berry growth and development (Fig. 10) [19, 20], the change of *VvDAO1* expression level in ‘Cabernet Sauvignon’ and ‘Pinot Noir’ is coincident with the IAA level, which is high in young berry, then decreases to a lower level before véraison (Fig. 6) [19]. This indicates *VvDAO1* may be responsible for the decrease of IAA level with the fruit development. As to the GA oxidase clades, *VvGA20oxs, VvGA3oxs* and *C*_*19*_*-VvGA2ox* are hardly expressed in fruits, while *C*_*19*_*-VvGA2ox* is responsible for GAs metabolism highly expressed in early development fruits (Fig. 6). This is consistent with the GA1 change pattern in Fig. 10 and another reported study—active GAs and their precursors in ‘Pinot Noir’ fruits could hardly be detected at fruit set stage (8 days after flowering) [29], the above results suggest *C*_*19*_*-VvGA2ox,* not *C*_*20*_*-VvGA2ox* participate in the berry growth by transforming active GA into inactive forms at fruit set stage.

However, what is more notable is the first investigation of the involvement of LBO, DMR6/DLO and JOX in berry development. The research of SLs functions mainly focuses on shoot branching [17], applying a synthetic strigolactone analogue, GR24, to wine grape fruit could influence the accumulation of sugar, anthocyanins and total phenols [46, 47], but the endogenous SLs level change patterns during berry development haven’t been studied yet. *VvLBO1* is expressed in fruit with a peak near véraison (Fig. 6), thus we speculate SLs and *VvLBO1* may be related to the onset of berry ripening. However, tomato *LBO* genes are expressed in roots and flowers while not in fruits [6]. It may be a good research point whether the expression difference of *LBOs* in grapes and tomatoes is related to the different ripening phenotypes. Endogenous SAs in grape fruit reached the highest level before the véraison stage, then gradually decreased and maintain a stable level until ripening (Fig. 10) [20]. Four *VvDMR6*/*DLO* genes are expressed in fruit, of which *VvDMR6b* and *VvDLO3* are highly expressed and up to the peak before véraison (Fig. 6), which may be responsible for the SAs level decrease at this stage [20]. Endogenous JA of grape fruit has a peak before véraison, then decreased sharply during véraison (Fig. 10) [20, 48]. *VvJOX5* expresses only at fruit set stage then close to 0 with berry growth, while *VvJOX6* is expressed highly during fruit development and reaches the peak later than the véraison stage (Fig. 6). The above analysis indicates, that it should be *VvJOX6* not *VvJOX5* maintains the low JAs level around véraison.

While the expression change of the above *Vv2OGDs* is consistent with the level of the hormones and they could be called major genes in each clade in berry, the others are not, such as *VvACO1, VvACO4b, VvDMR6a*, *VvDLO4,* etc. (Fig. 6). Do they functional and how they are functional, this may be an interesting direction in the next research.

### The response of *Vv2OGD-H* genes to MeJA treatment indicates the crosstalk of JAs-SAs, JAs-GAs and the negative feedback of endogenous JAs

JAs is usually considered a ripening inhibitor because of its lower level in mature berry than in early development berry (Fig. 10) [18, 19], MeJA application led to the decrease of total soluble solids and the increase of total acidity in *Vitis vinifera* cv. Sangiovese in recent research [24]. In this study, MeJA treatment was performed on ‘Jingxiangyu’ fruit twice around the véraison stage, one of the fruit maturity indexes — the total soluble solids [49] accumulated slower compared to control, which is consistent with the previous study [24], while the berry cluster volume, single berry weight and titratable acid have no significant differences compared to control (Fig. 7). This phenomenon indicates an inhibition function of exogenous MeJA in grape ripening process.

Most of the 16 *Vv2OGD-Hs* respond to MeJA treatment in ‘Jingxiangyu’ fruit (Fig. 8). In *Arabidopsis*, *JOX* is significantly up-regulated after exogenous MeJA treatment to maintain the endogenous JA level unchanged [13]. *VvJOX5* and *VvJOX6* also respond strongly to MeJA treatment in grape fruit (Fig. 8), which suggests the MeJA response mechanism in grape fruit may be similar to *Arabidopsis*. Both synergism and antagonism were reported in the interaction between JAs and ET [25]. In grape fruit, *VvACO1* and *VvACO4a* are slightly down-regulated after MeJA treatment, but *VvACO4b* is significantly up-regulated (Fig. 8). *VvACOs* may have a compensation mechanism in response to MeJA treatment to regulate ET release rate. JA and SA are antagonistic to each other in stress response as reported previously [25]. The SA metabolic enzymes *VvDMR6a*, *VvDLO3* and *VvDLO4* are up-regulated after MeJA treatment, only *VvDMR6b* was slightly down-regulated (Fig. 8). Therefore, the response mechanism of SAs to JAs in ripening process may be similar to the SAs-JAs antagonistic in stress-resistant process. In rice and *Arabidopsis*, GAs levels decreased under cold stress with the JA response gene expression, and JAZ (the inhibitor of JA response) regulated GAs levels positively [25]. The expression level of GA metabolic genes *VvGA2ox1a*, *VvGA2ox10* and *VvGA2ox11* are increased after MeJA treatment (Fig. 8), which also shows the antagonism of JAs-GAs interplay in grape fruit.

In summary, the response of the 16 *Vv2OGD-Hs* to MeJA treatment indicates the antagonism in JAs-SAs, JAs-GAs crosstalk, and the negative feedback of endogenous JAs after applying exogenous MeJA. These results are consistent with previous studies [13, 25, 50], and also reveal the conservation of hormone interaction mechanisms among different species and organs. Interestingly, GAs and SAs are generally considered ripening inhibitors [18, 19], however, the inhibition of the total soluble solid accumulation is accompanied by the up-regulation of SAs and GAs degrading-related enzymes (Fig. 7, Fig. 8), the functions of JAs-GAs and JAs-SAs crosstalk in grape fruit ripening process may be much complex and need further deeper research.

### The response of *Vv2OGD-H* genes to ABA treatment indicates the crosstalk of ABA with JAs, GAs, SAs and SLs

ABA was defined as a signal to trigger ripening process of grape fruit, and applying exogenous ABA hastens the ripening process [18, 19, 51]. In this study, the expression level of seven *Vv2OGD-H* genes changed significantly after ABA treatment in ‘Jingxiangyu’ fruits (Fig. 9). However, it is different from the previous studies [52] that the expression level of three *VvACOs* doesn’t show a significant increase, which may be due to the different varieties or treatment time. *VvLBO1* is significantly up-regulated after ABA treatment, which will cause the transformation of SLs into a more active form [MeCLA + 16 Da] [16]. Recently, the synergistic effect of ABA-SLs was reported in *Arabidopsis* and rice [26], but not in grapes until now. There even has been barely any research on SLs to fruit maturation. The response of *VvLBO1* to ABA treatment indicates the involvement of SLs and ABA-SLs interaction in grape ripening process. SA degrading genes *VvDLO3* and *VvDLO4* up-regulated remarkably after applying ABA (Fig. 9), which implies the antagonistic effect in ABA-SAs crosstalk. Similarly, SAs level in old leaves is decreased after the application of ABA in *Arabidopsis* [27]. The antagonistic effects of ABA-GAs are well known in different development stages and stress resistance processes, which partly result from the competition between endogenous ABA and GAs for the common precursor geranylgeranyl pyrophosphate (GGPP) [37]. GA degrading genes *VvGA2ox1a*, *VvGA2ox10* and *VvGA2ox11* are up-regulated after ABA treatment, while other members of *GA2ox* clade have no significant changes (Fig. 9), which may lead to less accumulation of GAs after ABA treatment. There has been much evidence that the crosstalk of ABA-JAs is synergistic, for example, the accumulation of JAs is accelerated after ABA treatment in *Arabidopsis* and rice [28]. In grape, JA metabolic gene *VvJOX6* is significantly down-regulated with ABA application (Fig. 9), which indicates there is also a synergistic effect between ABA and JAs in grape fruit.

In summary, the response of most *Vv2OGD-H* genes to exogenous ABA coincided with the reported hormone interaction previously—ABA could promote the synthesis of SLs and JAs (or inhibits their degradation), and inhibit the degradation of GAs and SAs [26–28, 37].

## Conclusions

In this study, 163 2OGD superfamily members in grapevine were identified, the family is mainly expanded by local (tandem and proximal) duplication, of which 37 members in 9 clades are related to the biosynthesis and metabolism of ET, SLs, IAA, SAs, GAs and JAs. Sixteen of the 37 *Vv2OGD-Hs* expressed in grape berry, in which *VvGA2oxs*, *VvDAOs* and *VvJOXs* may mainly cause the level change of GAs, IAA, JAs in berry. Moreover, eight *Vv2OGD-Hs* related to JAs, SAs and GAs metabolism are significantly up-regulated after MeJA treatment may be involved in the ripening inhibition of grape berry, while 7 *Vv2OGD-Hs* related to SLs, GAs, JAs, SAs biosynthesis and metabolism respond to ABA treatment may contribute to ripening promotion, which implies complex hormone interaction during fruit ripening. The 16 *Vv2OGD-Hs* expressed in grape berry and their response to MeJA or ABA treatment provides new insights into exploring the hormone regulation mechanism during grape berry development.

## Materials and methods

### Identification of Vv2OGD superfamily members

To identify 2OGD superfamily members in grape, the Pfam files of the 2OGD family were downloaded from the website [53], which include FeII_Oxy (PF03171), 2OG-FeII_Oxy_2 (PF13532), 2OG-FeII_Oxy_3 (PF13640), 2OG-FeII_Oxy_4 (PF13661) and 2OG-FeII_Oxy_5 (PF13759). Then these Pfam files were used to blast the grape protein database (https://phytozome-next.jgi.doe.gov/) by HMM search method in TBtools [54], the obtained members next were screened by removing different transcripts from one gene and whose e-value was greater than 0.0001, the screened members were uploaded to Pfam website and NCBI CD-search [55] to confirm it contains the 2OGD family domain.

### Construction of evolutional tree

The evolutionary tree was constructed by MEGA-X [56] with the amino acid sequence of the identified Vv2OGDs above and reported At2OGDs [3]. The construction method selected maximum likelihood, while the rest setting options were all default. Then the evolutionary tree was modified by iTOL [57]. Next, partial Vv2OGDs were named based on the evolutionary tree and published nomenclature [3], and the Vv2OGD-H members were identified according to the tree.

### Family expansion analysis and chromosome distribution of Vv2OGDs

The amino acid sequences of the grape genome were used for self-blast with E-value 1e^−5^. Then *Vv2OGDs* gene pairs of segment duplication and tandem duplication were obtained by uploading self-blast results and grape GFF files to TBtools [54]. The segment duplication results were displayed by Circos [58]. Chromosome distribution together with the tandem duplication analysis results was drawn by TBtools. Another two duplication ways proximal duplication and dispersed duplication were analyzed using the distance in chromosomes between *Vv2OGDs* based on the previous study [35].

### Collinearity analysis of Vv2OGDs-At2OGDs and Vv2OGDs-Sl2OGDs

The genome amino acid sequences of *Arabidopsis* [3], grape and tomato [6] were blasted to each other with E-value1e^−5^. Then the blast results were merged, and the GFF files of these three species were merged. Collinearity analysis was performed using TBtools [54] by inputting the merged blast results and GFF files, then gene pairs containing *Vv2OGDs* were selected and the final result was drawn by Circos [58].

### Sequence alignment and motif analysis

Amino acid sequences of hormone biosynthesis and metabolism related Vv2OGDs were used for sequence alignment and motif analysis. The sequence alignment was performed by the Clustal function in Jalview [59]. MEME website [41] was used for motif analysis, with parameters setting of motif number 50 and motif width 15, then the result was shown using TBtools.

### Published data used for pattern analysis of *Vv2OGD-Hs* expression and endogenous hormone

The expression level changes during grape berry development were investigated use transcriptome GSE98923 [33]. The transcriptome contains the fruit samples of ‘Pinot Noir’ and ‘Cabernet Sauvignon’ for three years (2012–2014). The detailed sampling information was described by Fasoli et al. [33]. In short, deseed samples were taken every 10 days from fruit set stage to fully mature stage in 2012, and every 7 days in 2013 and 2014. It is regarded as a meaningless expression if the RPKM value is less than 1, which means the gene is hardly expressed in this sample.

The changes in six endogenous hormones with the berry development were investigated in ‘Cabernet Sauvignon’ using the data from Gao et al. [20], Symons et al. [43], and Chervin et al. [42]. For the content of ABA, IAA, JA, SA and GA3, samples of pericarp, flesh and seed were taken every 10 days from DAF28 (days after flowering) to harvest in 2013 [20]. GA1 level was investigated in 2003 using the deseed samples taken from fruit set stage to harvest at DAF0, DAF14, DAF28, DAF56, DAF84, DAF112 [43]. The samples used for the changes of internal ET were taken from DAF35 to DAF 70 every week [42]. The diagram was generated using TBtools [54].

### The phenotype measure and qRT-PCR analysis after MeJA and ABA treatment

The *Vitis vinifera* cv ‘Jingxiangyu’ breeding by our laboratory was selected for MeJA and ABA treatment. The grape plants were planted in sunlight greenhouse of the grape germplasm nursery at Institute of Botany, the Chinese Academy of Sciences, which were cultivated by the coherent management of irrigation, fertilization, pruning and pest control. MeJA treatment performed in May 2021 as follows. Fruit clusters one week before the véraison stage (45 days after flowering) and in the same growth conditions were selected for the experiment. Three biological replicates were set for treatment and control groups, and 10 fruit clusters were randomly selected for each biological replicate. In the treatment group, fruit clusters were immersed in 10 mM MeJA solution (Shanghai Macklin, China) with 1% Tween-80 (Beijing Coolaber, China), while the control group was immersed in deionized water with 1% Tween-80. Seven days after the first treatment, the fruit entered the véraison stage (52 days after flowering), then the second treatment was performed in the same way as the first. For measuring the cluster volume, single berry weight, total soluble solids and titratable acid, samples were collected every one week from 7 days before treatment until fruit ripening (80 days after flowering). For the qRT-PCR experiment, samples were collected at 3 days, 0 days before treatment and 3 days, 7 days after each treatment. ABA treatment was performed exactly same as MeJA, except for the ABA concentration of 300 mg/L (Shanghai Macklin, China), samples for qRT-PCR were collected 3 days before treatment, 7 days after the first treatment, 3 and 7 days after the second treatment. After collection, the deseeded samples were frozen in liquid nitrogen immediately and stored in -80℃ refrigerator. Total RNA was extracted from collected samples using HiPure HP Plant RNA Mini Kit (R4165, Magen) followed the instructions. cDNA was synthesized by the kit of HiScript® III RT SuperMix for qPCR (R323, Vazyme). Then qRT-PCR was performed using AceQ® qPCR SYBR®Green Master Mix (Without ROX, Q121, Vazyme) followed its instruction with an Opticon thermocycler (CFX Connect Real-Time System; Bio-Rad, Hercules, CA). Primers used in qRT-PCR were listed in Suppl. Table S2, which was designed by NCBI primer-blast and synthesized in Beijing Tsingke Biotechnology Co., Ltd. The specificity of the primers was further determined by gel electrophoresis and sequencing. For each sample, three biological replicates and three technical replicates were performed. The relative expression level was figured out by Bio-Rad CFX Maestro software, *VvActin* (*VIT_204s0044g00580*) was used as a reference gene.

### Statistical analysis

All statistical analysis in this study was analyzed by T-test using SPSS 20, the mean and standard error from three biological repetitions was calculated by Excel, and the graphs were drawn by R software.

## Supplementary Information

**Additional file 1: Supplementary Figure S1.** Evolutionary tree of 163 Vv2OGDs and 130 At2OGDs. The branches in different clades are distinguished in different color. DOXA, DOXB and DOXC class are indicated at the outer arc, and different clades are indicated at the inside arc.

**Additional file 2: Supplementary Figure S2.** Sequence alignment of 37 Vv2OGD-H proteins. VvLBO3 is too long (701aa) to display, it is cut partly in C terminal. The locations of His-Xaa-Asp/Glu-(Xaa)n-His [HX(D/E)XnH] and Arg-Xaa-Ser/Thr (RxS/T) motif are highlighted in red and orange boxes respectively.

**Additional file 3: Supplementary Figure S3.** Motif analysis of 31 At2OGD-H and 37 Vv2OGD-H members. Phylogenetic tree (left) of 31 At2OGD-Hs and 37 Vv2OGD-Hs and their motif analysis (right). Different motifs are represented by different colored block with numbers in it. Sequence of each motif is listed in Suppl. Table S3.

**Additional file 4: Supplementary Table S1.** Detailed information of 163 members of Vv2OGD superfamily. **Supplementary Table S2.** Primers used in this study. **Supplementary Table S3.** Sequence and details of each motif in Suppl. Fig. S3.

## Data Availability

The Pfam files of the 2OGD family are available at [http://pfam.xfam.org/], the transcriptome GSE98923 are are available at [https://www.ncbi.nlm.nih.gov/geo/query/acc.cgi?acc=GSE98923]. The datasets and materials used during the current study are available from the corresponding author on reasonable request.
