# Correction: Hormone biosynthesis and metabolism members of 2OGD superfamily are involved in berry development and respond to MeJA and ABA treatment of *Vitis vinifera* L

**DOI:** 10.1186/s12870-022-03863-8

**Published:** 2022-10-10

**Authors:** Gao Yingying, Wang Xiaochen, Xianju Liu, Zhenchang Liang

**Affiliations:** 1 Beijing Key Laboratory of Grape Science and Enology, CAS Key Laboratory of Plant Resources, Institute of Botany, The Chinese Academy of Sciences, 100093 Beijing, China; 2 University of Chinese Academy of Sciences, 100049 Beijing, China; 3 Key Laboratory of Beijing for Identification and Safety Evaluation of Chinese Medicine, Institute of Chinese Materia Medica, China Academy of Chinese Medical Sciences, 100700 Beijing, China

**Correction:** ***BMC Plant Biol*** **22, 427 (2022)**

10.1186/s12870-022-03810-7

Following publication of the original article [1], an error was identified in the presentation of the two authors. Gao Yingying and Wang Xiaochen should be presented as Yingying Gao and Xiaochen Wang.

Given name: Yingying

Family name: Gao

Given name: Xiaochen

Family name: Wang

The correction does not have any effect on the results or conclusions of the paper. The original article has been corrected.

## References

[1] Gao Y, Wang X, Liu X (2022). Hormone biosynthesis and metabolism members of 2OGD superfamily are involved in berry development and respond to MeJA and ABA treatment of *Vitis vinifera* L. BMC Plant Biol.

