# Supplementary figures and images for: Hormone biosynthesis and metabolism members of 2OGD superfamily are involved in berry development and respond to MeJA and ABA treatment of Vitis vinifera L
Source: BMC Plant Biol. 2022 Sep 6;22:427. doi: 10.1186/s12870-022-03810-7 (PMC9446723; doi:10.1186/s12870-022-03810-7)

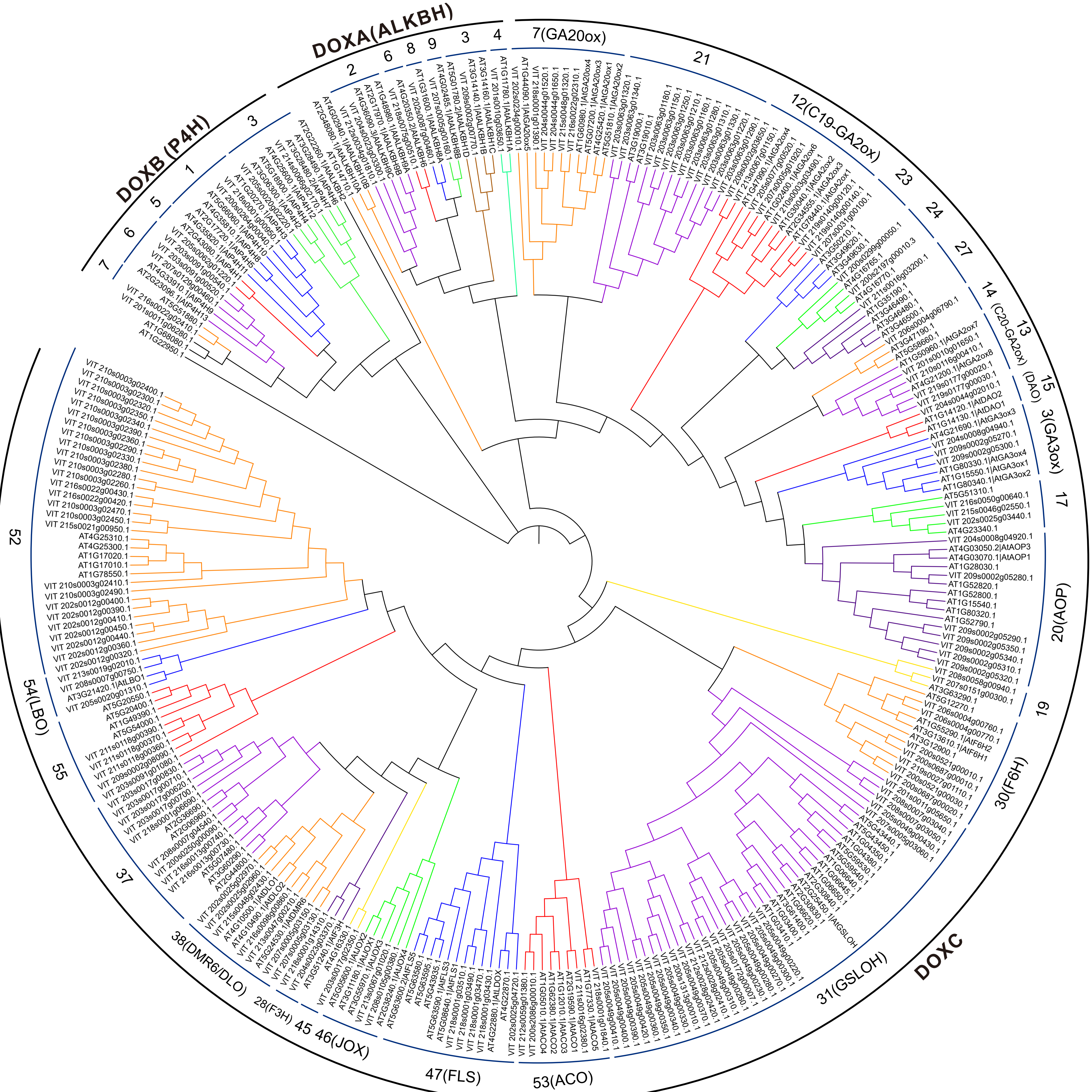

Supplement: Supplementary file 1 — Additional file 1: Supplementary Figure S1. Evolutionary tree of 163 Vv2OGDs and 130 At2OGDs. The branches in different clades are distinguished in different color. DOXA, DOXB and DOXC class are indicated at the outer arc, and different clades are indicated at the inside arc. [file 12870_2022_3810_MOESM1_ESM.pdf]

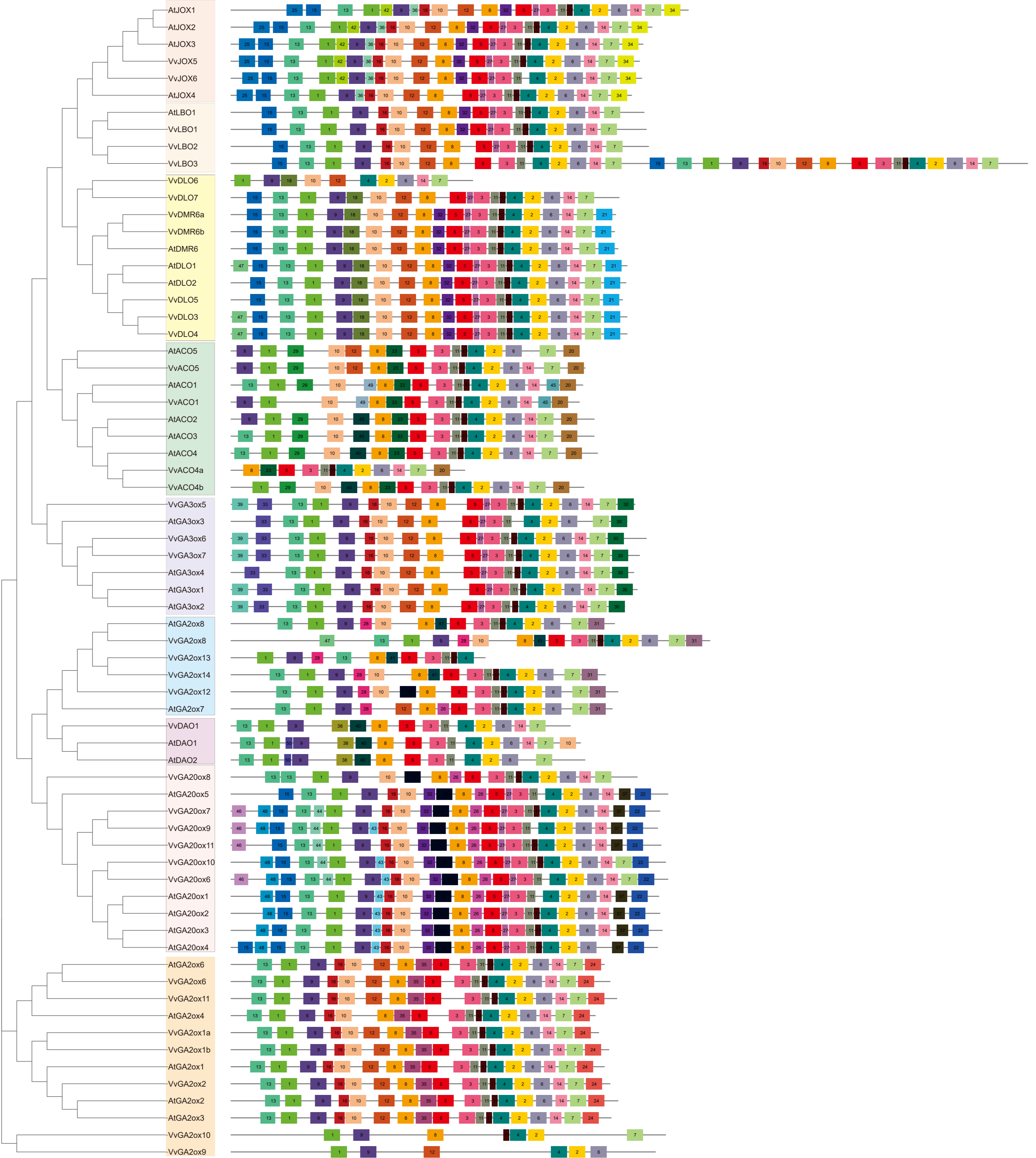

Supplement: Supplementary file 3 — Additional file 3: Supplementary Figure S3. Motif analysis of 31 At2OGD-H and 37 Vv2OGD-H members. Phylogenetic tree (left) of 31 At2OGD-Hs and 37 Vv2OGD-Hs and their motif analysis (right). Different motifs are represented by different colored block with numbers in it. Sequence of each motif is listed in Suppl. Table S3. [file 12870_2022_3810_MOESM3_ESM.pdf]
