# Supplementary material for: Hormone biosynthesis and metabolism members of 2OGD superfamily are involved in berry development and respond to MeJA and ABA treatment of Vitis vinifera L
Source: BMC Plant Biol. 2022 Sep 6;22:427. doi: 10.1186/s12870-022-03810-7 (PMC9446723; doi:10.1186/s12870-022-03810-7)
Supplement: Supplementary file 2 — Additional file 2: Supplementary Figure S2. Sequence alignment of 37 Vv2OGD-H proteins. VvLBO3 is too long (701aa) to display, it is cut partly in C terminal. The locations of His-Xaa-Asp/Glu-(Xaa)n-His [HX(D/E)XnH] and Arg-Xaa-Ser/Thr (RxS/T) motif are highlighted in red and orange boxes respectively. [file 12870_2022_3810_MOESM2_ESM.pdf]

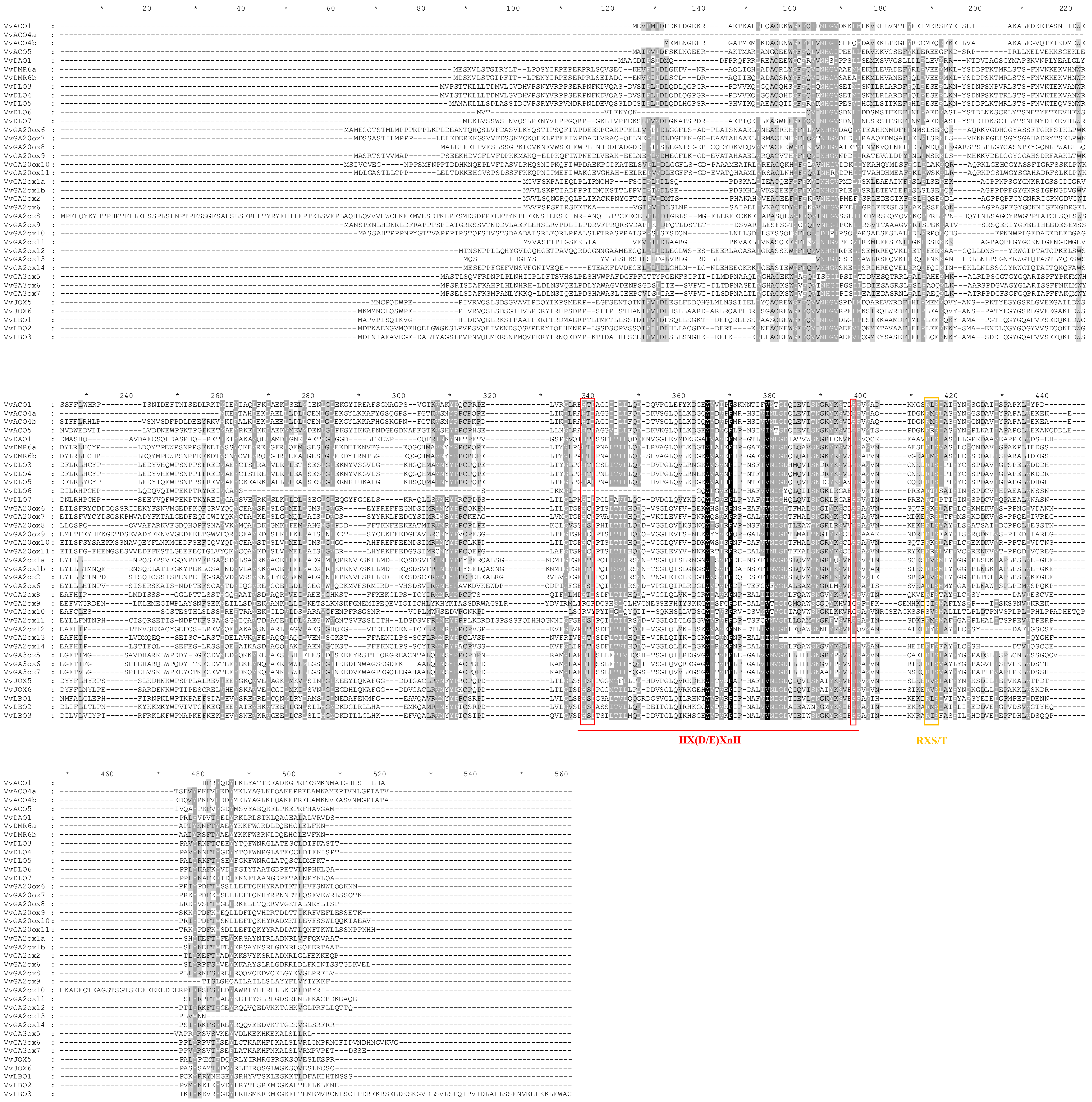
HX(D/E)XnH
RXS/T
